# Association of dietary phytochemical index with sleep quality, and inflammatory markers in adults with type 2 diabetes: a cross-sectional study

**DOI:** 10.3389/fnut.2026.1766495

**Published:** 2026-04-07

**Authors:** Shuailong Li, Yixing Li, Xiaowei Yu, Zhixian Liu, Long Li, Jingjing Liu, Haibin Yuan, Qiang Luo

**Affiliations:** 1 Department of Anesthesia, The First People’s Hospital of Xiangtan City, Xiangtan, Hunan Province, China; 2 Cancer Research Center, The First People’s Hospital of Xiangtan City, Xiangtan, Hunan Province, China; 3 The First People’s Hospital of Xiangtan City, Xiangtan, Hunan Province, China; 4 Department of Endocrinology, Pingxiang Maternal and Child Health Hospital, Pingxiang, Jiangxi Province, China; 5 Department of Anesthesia, Xiangxi Tujia and Miao Autonomous Prefecture People’s Hospital, Jishou, Hunan Province, China

**Keywords:** dietary phytochemical index, gutmicrobiota, inflammation, sleep quality, type 2 diabetes mellitus

## Abstract

**Background:**

Type 2 diabetes mellitus (T2DM) is associated with sleep disturbances, inflammation, and gut dysbiosis, potentially modifiable by diet. This study examined associations between the dietary phytochemical index (DPI), sleep quality, systemic inflammation, and oxidative stress markers in adults with T2DM.

**Methods:**

In this cross-sectional study, 675 adults with T2DM (aged 35–75 years) were recruited. DPI was calculated from a validated Food Frequency Questionnaire. Objective sleep was assessed via BodyMedia SenseWear armband (duration, efficiency, latency, WASO); subjective sleep via Pittsburgh Sleep Quality Index (PSQI). Inflammatory markers (CRP, IL-6, TNF-α), oxidative stress (MDA, TAC, SOD), and hormones (melatonin, cortisol) were measured. Linear regression and mediation analyses were performed.

**Results:**

Participants in the highest DPI quartile (Q4) had longer sleep duration (422.69 ± 20.01 vs. 367.47 ± 43.36 min, *p* < 0.001), shorter sleep latency (11.9 ± 2.47 vs. 19.1 ± 7.8 min, *p* < 0.001), lower wake-after-sleep-onset (39.44 ± 4.09 vs. 52.3 ± 9.64 min, *p* < 0.001), higher sleep efficiency (90.21 ± 3.14 vs. 84.83 ± 6.68%, *p* < 0.001), and lower PSQI scores (4.49 ± 1.1 vs. 6.81 ± 1.5, *p* < 0.001) compared with Q1. Inflammatory markers were lower in Q4: CRP (1.90 ± 0.40 vs. 3.60 ± 0.90 mg/L), IL-6 (3.28 ± 0.58 vs. 5.07 ± 1.02 pg./mL), and TNF-*α* (4.71 ± 0.55 vs. 6.59 ± 1.13 pg./mL, all *p* < 0.001). Higher DPI was also associated with more favorable oxidative stress profiles and hormone concentrations.

**Conclusion:**

Higher DPI scores is associated with better sleep quality, and lower systemic inflammation in adults with T2DM. These findings highlight the potential role of phytochemical-rich diets in supporting sleep quality and metabolic health among adults with type 2 diabetes; however, prospective randomized controlled trials are required to confirm these associations and to establish causality.

## Introduction

Type 2 diabetes mellitus (T2DM) is a chronic metabolic disorder characterized by insulin resistance, hyperglycemia, and low-grade systemic inflammation (1). Sleep disturbances are common among adults with T2DM and have been linked to impaired glycemic control, increased inflammatory markers, and higher risk of cardiovascular and metabolic complications (2, 3). Evidence suggests that diet can influence both sleep quality and metabolic health, yet the role of specific dietary components, particularly plant-derived bioactive compounds, remains underexplored in this population (4–7).

The dietary phytochemical index (DPI) is a measure of habitual intake of phytochemical-rich foods, including fruits, vegetables, whole grains, legumes, nuts, seeds, and minimally processed plant-based products (8) Because DPI is calculated as the proportion of daily energy from plant foods, it inherently correlates with overall dietary quality, fiber intake, and multiple dietary components. Therefore, DPI cannot isolate phytochemical-specific effects independent from other beneficial components of plant-based diets, including fiber, vitamins, minerals, and overall dietary pattern quality. Phytochemicals, such as polyphenols, flavonoids, and carotenoids, exert anti-inflammatory, antioxidant, and metabolic regulatory effects (9). Previous studies have linked higher dietary phytochemical intake to improved cardiovascular outcomes, reduced oxidative stress, and lower systemic inflammation (10, 11). However, evidence regarding the relationship between DPI and sleep quality is limited, especially among individuals with T2DM (12). While some research has examined overall dietary patterns—such as Mediterranean or plant-based diets—in relation to sleep, the specific contribution of phytochemicals remains unclear (13–15).

Understanding these relationships is particularly relevant in T2DM, where impaired glucose metabolism, inflammation, and sleep disturbances interact to exacerbate disease progression and complications (16). Investigating dietary phytochemicals in relation to both objective and subjective sleep measures, and inflammatory markers may provide actionable insights for nutritional strategies aimed at improving sleep and metabolic health.

Therefore, the present study aimed to examine the associations between DPI, sleep quality, and systemic inflammation in adults with T2DM.

## Methods

### Study design and participants

**Figure 1:**
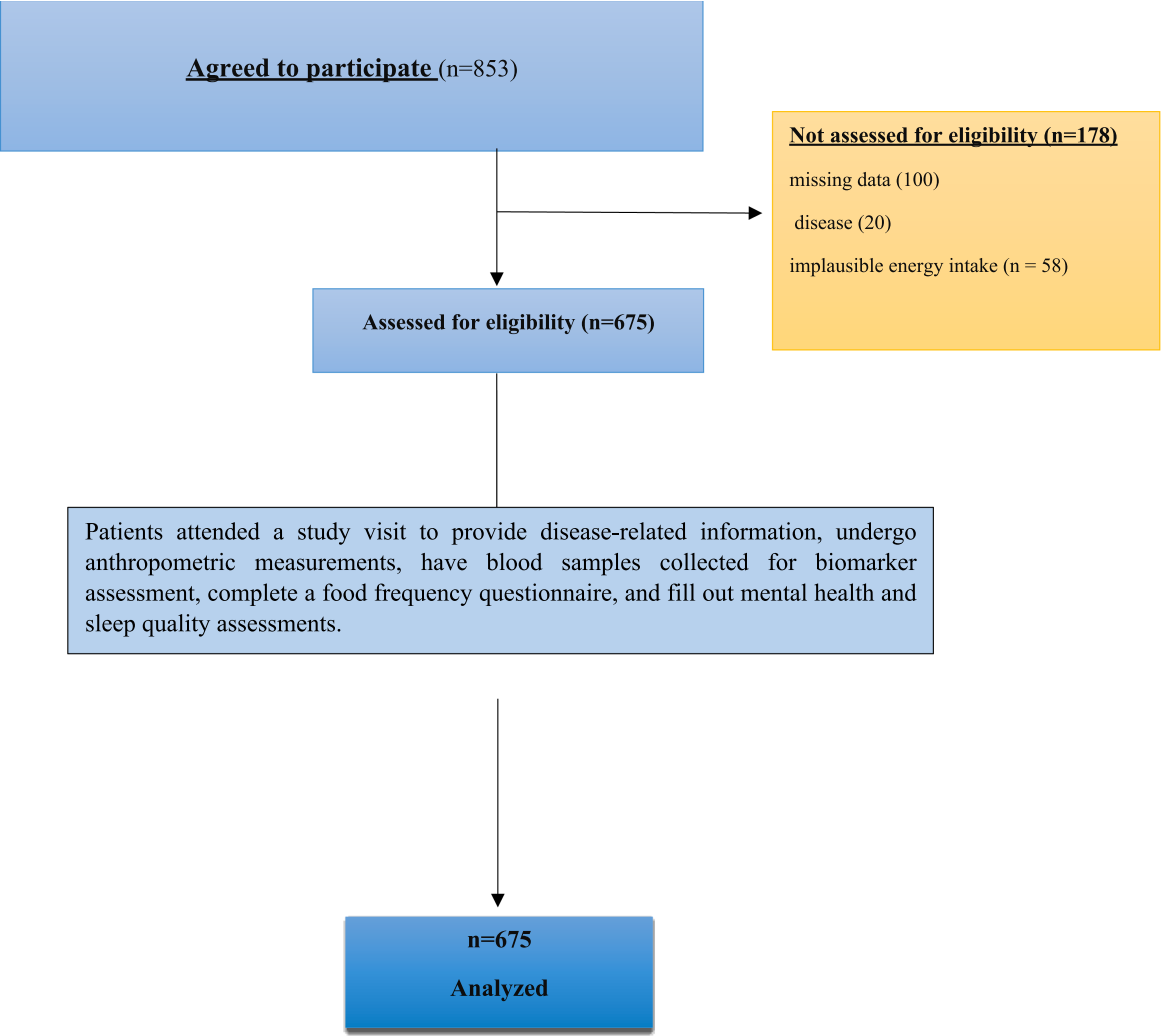
Flowchart of study participants.

This cross-sectional study was conducted among adults diagnosed with T2DM. A total of 853 individuals were initially recruited. Of these, 178 participants were excluded due to incomplete questionnaire responses (*n* = 67), the presence of chronic diseases other than type 2 diabetes (*n* = 20), “implausible reported energy intake (<1,000 or >5,000 kcal/day) (*n* = 58), and age <35 years (*n* = 33). Consequently, 675 participants were included in the final analytical sample (Figure 1). Participants were recruited consecutively from outpatient diabetes clinics in the Pingxiang Maternal and Child Health Hospital, China. Eligible individuals were aged 35–75 years, had a confirmed diagnosis of type 2 diabetes for at least 1 year, and had no acute or chronic conditions that could influence sleep, inflammatory status, or gut microbiota (e.g., active infection, cancer, autoimmune disorders, gastrointestinal surgery). Individuals who had used antibiotics, prebiotics, probiotics, corticosteroids, or sleep medications within the past three months were excluded.

Data collection was conducted through face-to-face interviews using a structured questionnaire administered by trained researchers. The questionnaire captured demographic and lifestyle information, dietary assessment, anthropometric measurements, subjective sleep quality using the Pittsburgh Sleep Quality Index (PSQI), physical activity using the International Physical Activity Questionnaire (IPAQ), and dietary intake through a 24-h dietary recall. To minimize bias, validated instruments were used for all assessments, standardized protocols were followed for anthropometric and biochemical measurements, and trained staff conducted interviews to reduce interviewer variability.

All participants provided written informed consent before enrollment. Ethical approval was obtained from the Pingxiang Maternal and Child Health Hospital (Approval No. 20250908033), and the study was conducted in accordance with the principles of the Declaration of Helsinki.

### Sample size calculation

The required sample size was calculated using G*Power software. Assuming a two-sided significance level (*α* = 0.05), statistical power of 80%, and an expected small-to-moderate effect size (Cohen’s d = 0.3) for differences in sleep quality across DPI quartiles, the minimum sample size was estimated at 575 participants (17). To account for potential exclusions and missing data, we aimed to recruit at least 675 individuals. Ultimately, 675 participants were included in the final analysis. The calculation was specifically designed to detect differences in sleep quality across DPI quartiles, which represented the primary study objective.

### Dietary assessment and calculation of the dietary phytochemical index (DPI)

Dietary intake was assessed using a validated Food Frequency Questionnaire (FFQ) reflecting habitual consumption over the past year. To minimize recall bias—especially in older participants—dietary data were collected using interviewer-administered questionnaires, supported by visual aids, and interviews were conducted by trained dietitians. Nutrient composition was calculated using the Chinese Food Composition Tables. The DPI was calculated as the percentage of daily energy derived from phytochemical-rich foods relative to the total daily energy intake. Phytochemical-rich foods included fruits, vegetables, whole grains, legumes, nuts, seeds, and minimally processed plant-based items, with coffee, tea, spices, and olive oil included according to established DPI definitions. Total energy intake, macronutrients (protein, carbohydrate, fat), fiber, fruit and vegetable intake, legumes, nuts, dairy, and whole grains were included as dietary covariates in fully adjusted models. In other words, $\mathrm{DPI}=(\begin{array}{l} \text{daily energy from phytochemical}-\\ \text{rich foods}÷\text{total daily energy intake} \end{array})\times 100$ .

### Anthropometric and general measures

Trained staff measured weight, height, waist, and hip circumferences following standardized protocols, and BMI was calculated as kg/m^2^. Blood pressure was recorded using an automated sphygmomanometer after a 10-min seated rest. Additional variables—including socioeconomic status (SES), smoking status, diabetes duration, and medication use (including oral hypoglycemic agents, insulin, antihypertensive medications, and lipid-lowering drugs)—were collected via structured interview. Physical activity was evaluated using the IPAQ-Short Form, yielding MET-minutes/week.

### Assessment of sleep parameters

#### Objective sleep assessment

Objective sleep indicators were measured using the BodyMedia SenseWear^®^ armband, a validated multisensor device for free-living conditions. The armband integrates heat-flux sensors, galvanic skin response detectors, a triaxial accelerometer, and a thermistor-based skin surface sensor to monitor sleep–wake patterns (6). Participants wore the armband continuously for 7 consecutive nights, and average values were calculated for analysis. The device quantified key sleep parameters, including sleep latency (time to fall asleep), wake-after-sleep-onset (WASO), nighttime sleep duration, and sleep efficiency. Sleep efficiency was calculated as the ratio of nighttime sleep duration to the sum of sleep duration, sleep latency, and WASO, reflecting the proportion of time spent asleep relative to total time in bed. Validation studies have demonstrated high agreement with actigraphy for estimating sleep duration, WASO, and sleep efficiency.

#### Subjective sleep assessment (PSQI)

Subjective sleep quality was assessed using the Pittsburgh Sleep Quality Index (PSQI), which includes 19 self-rated items across seven components: subjective sleep quality, sleep latency, sleep duration, habitual sleep efficiency, sleep disturbances, use of sleep medication, and daytime dysfunction. Component scores were summed to yield a global score ranging from 0 to 21, with higher scores indicating poorer sleep quality. A global PSQI score >5 was considered indicative of poor sleep quality, consistent with established cut-points (18, 19).

### Assessment of anxiety and depression

Psychological variables were included due to the established link between sleep behavior and mental well-being. Anxiety symptoms were measured using the Generalized Anxiety Disorder 7-item Scale (GAD-7), which includes seven items scored on a 4-point Likert scale (0 = “not at all” to 3 = “nearly every day”), yielding a total score of 0–21; scores ≥10 indicated moderate to severe anxiety. Depressive symptoms were measured using the Patient Health Questionnaire-9 (PHQ-9), with nine items scored similarly, yielding a total score of 0–27; scores ≥10 indicated moderate or higher depressive symptoms. Both instruments have been validated across diverse populations and settings (20).

### Gut permeability and microbial metabolites

Intestinal permeability was assessed by measuring serum zonulin using ELISA kits. Microbial metabolites—including trimethylamine N-oxide (TMAO), indole derivatives, and short-chain fatty acids (acetate, propionate, butyrate)—were analyzed using gas chromatography–mass spectrometry (GC–MS) with validated standards.

#### Biochemical assessments

Following a 10–12 h overnight fast, venous blood samples were collected, and plasma and serum were separated and stored at −80 °C. Inflammatory markers—CRP, IL-6, TNF-*α*, and LPS—were measured using commercially available ELISA kits (e.g., R&D Systems, Abcam, Cusabio). Oxidative stress markers were measured with MDA (colorimetric assay, Cayman Chemical), total antioxidant capacity (TAC, Sigma-Aldrich), and superoxide dismutase (SOD, Cayman Chemical).

Metabolic indicators included fasting glucose (enzymatic colorimetric kits, Roche Diagnostics), HbA1c (ELISA, Abcam), fasting insulin (immunoassay, Mercodia), and HOMA-IR calculated from glucose and insulin. Lipid profiles—total cholesterol, LDL-C, HDL-C, triglycerides—were assessed using enzymatic colorimetric kits (Randox, Roche).

Melatonin and cortisol were quantified using high-sensitivity ELISA kits (IBL International, Enzo Life Sciences) from morning blood samples collected between 07:00 and 09:00 after overnight fasting. These represent single time-point circulating concentrations and do not allow inference about diurnal profiles or circadian phase.

### Statistical analysis

All analyses were performed following STROBE guidelines. All statistical analyses were performed using SPSS version 27.0, IBM Corp. Statistical significance was set at *p* < 0.05 for all tests except where Bonferroni correction was applied. DPI scores were categorized into quartiles (Q1–Q4). Continuous variables were expressed as mean ± SD and categorical variables as frequencies and percentages. Differences across DPI quartiles were assessed using ANOVA for continuous variables and chi-square tests for categorical variables.

Associations between DPI and outcomes—including sleep parameters, inflammatory markers, oxidative stress indicators, and hormonal variables were evaluated using three models.

Associations between DPI and outcomes—including sleep parameters, inflammatory markers, oxidative stress indicators, and hormonal variables—were evaluated using three models. Primary confirmatory outcomes were associations of DPI with: (1) sleep quality (PSQI global score, objective duration, efficiency, latency, WASO), (2) key inflammatory markers (CRP, IL-6, TNF-*α*), and (3) oxidative stress markers (MDA, TAC, SOD). Bonferroni correction was applied to the primary outcomes (α = 0.05/10 = 0.005).

## Results

### Participant characteristics and dietary intake

The study included 675 adults with T2DM, with a mean age of 60.1 ± 2.9 years; 48.1% were female (Table 1). Participants were categorized into quartiles of the DPI: Q1 (lowest) to Q4 (highest). Age, diabetes duration, and physical activity did not differ significantly across DPI quartiles (all *p* > 0.05), indicating comparable demographic and lifestyle characteristics among groups. However, the proportion of female participants (*p* = 0.011), socio-economic status (*p* = 0.018), and smoking prevalence varied across quartiles, suggesting some distributional differences in demographic and lifestyle factors.

**Table 1:** Baseline characteristics and dietary intake across quartiles of the dietary phytochemical index (DPI) in adults with type 2 diabetes (*N* = 675).

Variables	Total (*N* = 675)	Q1 (*N* = 180)	Q2 (*N* = 171)	Q3 (*N* = 173)	Q4 (*N* = 151)	*P* value
Age (years)	60.13 ± 2.87	59.86 ± 3.30	60.27 ± 1.90	59.96 ± 3.69	60.47 ± 2.04	0.161
Sex, female, *n* (%)	325 (100%)	72 (22.2%)	81 (24.9%)	84 (25.8%)	88 (27.1%)	0.011
Socio-Economic Status (SES), *n* (%)						0.540
Low	248 (100%)	60 (24.2%)	72 (29.0%)	60 (24.2%)	56 (22.6%)	
Medium	150 (100%)	37 (24.7%)	38 (25.3%)	40 (26.7%)	35 (23.3%)	
High	274 (100%)	82 (29.9%)	60 (21.9%)	73 (26.6%)	59 (21.5%)	
Smoking, *n* (%)	62 (100%)	17 (27.4%)	21 (33.9%)	6 (9.7%)	18 (29.0%)	0.018
Diabetes duration (years)	5.76 ± 1.32	5.95 ± 1.12	5.46 ± 1.56	5.96 ± 1.57	5.66 ± 0.71	0.083
DPI	38.47 ± 8.91	27.53 ± 5.38	36.11 ± 1.96	42.42 ± 1.63	49.68 ± 4.50	<0.001
BMI (kg/m^2^)	28.71 ± 1.62	30.11 ± 1.51	29.37 ± 0.92	28.22 ± 0.99	26.84 ± 0.52	<0.001
WC (cm)	86.40 ± 4.86	90.63 ± 4.54	88.39 ± 2.78	84.93 ± 2.97	80.79 ± 1.58	<0.001
Physical activity (MET)	1528.82 ± 108.28	1536.33 ± 97.93	1523.30 ± 124.22	1529.19 ± 130.89	1525.70 ± 63.16	0.784
Energy intake (kcal/day)	2022.84 ± 76.04	2096.14 ± 78.07	2025.44 ± 71.57	1971.18 ± 30.16	1991.72 ± 36.81	<0.001
Carbohydrate (g/day)	237.80 ± 13.92	249.21 ± 15.37	237.11 ± 12.99	230.31 ± 3.72	233.58 ± 11.94	<0.001
Protein (g/day)	87.45 ± 3.94	86.28 ± 4.88	85.68 ± 3.27	88.73 ± 2.85	89.37 ± 3.04	0.002
Fat (g/day)	70.78 ± 4.93	75.71 ± 5.14	71.47 ± 3.68	67.57 ± 2.20	67.79 ± 2.44	<0.001
Fiber (g/day)	25.96 ± 5.35	21.52 ± 5.42	23.05 ± 1.97	28.00 ± 1.65	32.22 ± 2.90	<0.001
Fruits & Vegetables (g/day)	427.54 ± 62.96	360.43 ± 54.07	402.61 ± 32.05	460.15 ± 17.76	498.42 ± 20.08	<0.001
Legumes (g/day)	43.60 ± 11.61	31.49 ± 9.75	38.23 ± 5.08	49.62 ± 3.97	57.20 ± 2.79	<0.001
Whole grains (g/day)	148.71 ± 31.28	121.67 ± 30.21	131.39 ± 11.35	161.90 ± 10.88	185.45 ± 15.71	<0.001
Milk (mL/day)	238.96 ± 20.31	224.63 ± 21.80	229.05 ± 6.42	241.58 ± 7.34	264.24 ± 12.96	<0.001
Nuts (g/day)	20.59 ± 5.77	15.41 ± 5.76	17.74 ± 2.05	22.76 ± 1.78	27.54 ± 2.47	<0.001

Values are presented as mean ± standard deviation (SD) for continuous variables and number (percentage) for categorical variables. DPI quartiles were defined as: Q1 (lowest), Q2, Q3, and Q4 (highest). *P*-values were obtained using ANOVA for continuous variables and Chi-square tests for categorical variables.

As expected, DPI values increased monotonically across quartiles (Q1: 27.5 ± 5.4 vs. Q4: 49.7 ± 4.5; *p* < 0.001). Higher DPI was associated with lower BMI (Q1: 30.1 ± 1.5 vs. Q4: 26.8 ± 0.5 kg/m^2^) and waist circumference, reflecting a potential link between higher phytochemical intake and anthropometric profiles. Total energy intake showed a slight decrease with higher DPI, whereas intake of protein, dietary fiber, fruits and vegetables, legumes, whole grains, milk, and nuts increased substantially across quartiles (all *p* < 0.01), demonstrating distinct dietary patterns among participants with varying DPI scores.

### Inflammatory, oxidative stress, glycemic, and lipid, hormonal

Participants in higher DPI quartiles exhibited lower levels of inflammation and oxidative stress markers, including CRP (Q1: 3.60 ± 0.90 vs. Q4: 1.90 ± 0.40 mg/L), IL-6 (5.07 ± 1.02 vs. 3.28 ± 0.58 pg./mL), TNF-*α*, LPS, and MDA (all *p* < 0.001; Table 2). In contrast, antioxidant markers such as TAC and SOD increased progressively across quartiles, indicating more favorable oxidative stress profiles with higher DPI.

**Table 2:** Inflammatory, oxidative stress, glycemic, lipid, and hormonal biomarkers across quartiles of the dietary phytochemical index (DPI) (*N* = 675).

Variables	Total (*N* = 675)	Q1 (*N* = 180)	Q2 (*N* = 171)	Q3 (*N* = 173)	Q4 (*N* = 151)	*P* value
CRP (mg/L)	2.91 ± 0.85	3.60 ± 0.90	3.32 ± 0.43	2.67 ± 0.28	1.90 ± 0.40	<0.001
IL6 (pg./mL)	4.29 ± 0.93	5.07 ± 1.02	4.66 ± 0.44	4.00 ± 0.31	3.28 ± 0.58	<0.001
TNFα (pg./mL)	5.73 ± 1.02	6.59 ± 1.13	6.09 ± 0.65	5.38 ± 0.31	4.71 ± 0.55	<0.001
LPS (EU/mL)	0.56 ± 0.12	0.67 ± 0.12	0.61 ± 0.06	0.52 ± 0.05	0.43 ± 0.06	<0.001
MDA (μmol/L)	2.60 ± 0.67	3.16 ± 0.71	2.87 ± 0.37	2.41 ± 0.22	1.85 ± 0.33	<0.001
TAC (mmol/L)	1.66 ± 0.38	1.36 ± 0.41	1.51 ± 0.13	1.73 ± 0.09	2.11 ± 0.28	<0.001
SOD (U/mL)	125.71 ± 18.91	108.73 ± 18.88	117.70 ± 4.84	130.73 ± 5.27	149.11 ± 10.87	<0.001
Fasting glucose (mg/dL)	122.67 ± 15.64	135.97 ± 12.58	130.57 ± 9.71	117.32 ± 9.87	114.16 ± 5.33	<0.001
HbA1c (%)	7.13 ± 0.86	7.71 ± 0.96	7.54 ± 0.52	6.95 ± 0.32	6.67 ± 0.46	<0.001
HOMA-IR	2.74 ± 0.74	3.29 ± 0.75	3.13 ± 0.28	2.57 ± 0.36	2.31 ± 0.35	<0.001
Total cholesterol (mg/dL)	178.16 ± 18.32	191.30 ± 17.45	187.36 ± 12.29	173.56 ± 12.16	157.54 ± 6.78	<0.001
Triglycerides (mg/dL)	145.90 ± 20.73	164.02 ± 17.51	155.25 ± 14.31	138.12 ± 12.58	122.82 ± 7.01	<0.001
HDL (mg/dL)	53.50 ± 9.18	47.33 ± 10.11	49.98 ± 1.46	53.74 ± 1.88	64.51 ± 8.10	<0.001
LDL (mg/dL)	106.63 ± 17.42	122.16 ± 15.85	114.23 ± 11.99	99.84 ± 8.54	87.29 ± 5.07	<0.001
Cortisol (nmol/L)	368.74 ± 29.32	396.11 ± 23.43	380.86 ± 20.39	355.11 ± 18.67	337.97 ± 9.59	<0.001
Melatonin (pg./mL)	25.74 ± 8.83	19.22 ± 9.40	21.71 ± 2.64	26.79 ± 2.33	28.79 ± 5.85	<0.001
TMAO (μmol/L)	2.92 ± 0.91	3.63 ± 0.97	3.35 ± 0.57	2.64 ± 0.32	1.90 ± 0.38	<0.001
Acetate (μmol/L)	49.74 ± 13.17	40.46 ± 13.79	43.77 ± 3.71	50.59 ± 3.91	66.50 ± 9.28	<0.001
Propionate (μmol/L)	32.52 ± 10.75	25.48 ± 12.56	29.00 ± 2.50	32.71 ± 2.25	44.62 ± 9.64	<0.001
Butyrate (μmol/L)	21.61 ± 8.10	16.37 ± 9.31	18.32 ± 2.23	22.11 ± 2.25	30.94 ± 6.67	<0.001
Zonulin (ng/mL)	51.27 ± 14.74	63.49 ± 13.70	58.32 ± 10.06	46.54 ± 5.22	34.18 ± 6.79	<0.001

Values are expressed as mean ± SD. Between-group differences were evaluated using one-way ANOVA. CRP, C-reactive protein; IL-6, interleukin-6; TNF-α, tumor necrosis factor-alpha; LPS, lipopolysaccharide; MDA, malondialdehyde; TAC, total antioxidant capacity; SOD, superoxide dismutase; HbA1c, glycated hemoglobin; HDL, high-density lipoprotein; LDL, low-density lipoprotein; TMAO, trimethylamine-N-oxide; SCFAs, short-chain fatty acids.

Higher DPI was associated with better glycemic indices, including fasting glucose (135.97 ± 12.6 vs. 114.2 ± 5.3 mg/dL), HbA1c (7.71 ± 0.96 vs. 6.67 ± 0.46%), and HOMA-IR (3.29 ± 0.75). Favorable lipid profiles were also observed, with lower total cholesterol, triglycerides, LDL, and higher HDL concentrations in participants with higher DPI. Fasting hormone concentrations differed across DPI quartiles. Melatonin concentrations measured at a single fasting timepoint were higher in participants with higher DPI (Q1: 19.22 ± 9.40 vs. Q4: 28.79 ± 5.85 pg./mL), and cortisol concentrations were lower (Q1: 396.11 ± 23.43 vs. Q4: 337.97 ± 9.59 nmol/L). These single-timepoint measurements reflect associations with circulating hormone levels but cannot characterize circadian rhythms or sleep–wake regulation.

Gut microbial metabolite differed across DPI quartiles, with higher DPI associated with higher SCFAs (acetate, propionate, butyrate). The zonulin levels was lower with higher DPI (all *p* < 0.001), suggesting more favorable gut microbial and barrier profiles in participants with higher dietary phytochemical intake (Table 2).

### Sleep duration and sleep quality

Sleep duration and sleep quality parameters varied across DPI quartiles (Table 3). Participants in the highest DPI quartile (Q4) reported longer average sleep duration (422.7 ± 20.0 min) than those in the lowest quartile (Q1: 367.5 ± 43.4 min; *p* < 0.001). Sleep efficiency was also higher in Q4 (90.2 ± 3.1%) compared with Q1 (84.8 ± 6.7%; *p* < 0.001). Global PSQI scores were lower among individuals in higher DPI quartiles, with values ranging from 6.81 ± 1.70 in Q1 to 4.49 ± 0.60 in Q4 (*p* < 0.001). Indicators of sleep continuity showed a similar pattern. Wake after sleep onset (WASO) ranged from 52.0 ± 9.6 min in Q1 to 39.4 ± 4.1 min in Q4, and sleep latency ranged from 19.0 ± 7.8 min to 11.9 ± 2.5 min across the same quartiles (both *p* < 0.001). Overall, participants in higher DPI quartiles demonstrated longer sleep duration, higher sleep efficiency, and more favorable sleep quality indices.

**Table 3:** Sleep duration and sleep quality parameters across quartiles of the dietary phytochemical index (DPI) (*N* = 675).

Variables	Total (*N* = 675)	Q1 (*N* = 180)	Q2 (*N* = 171)	Q3 (*N* = 173)	Q4 (*N* = 151)	*P* value
Sleep duration (min)	402.71 ± 37.04	367.47 ± 43.36	394.56 ± 20.46	429.99 ± 13.90	422.69 ± 20.01	<0.001
PSQI	5.76 ± 1.38	6.81 ± 1.70	6.35 ± 0.61	5.20 ± 0.76	4.49 ± 0.60	<0.001
Sleep latency (min)	16.03 ± 5.87	19.01 ± 7.80	19.16 ± 4.54	13.44 ± 2.26	11.90 ± 2.47	<0.001
Sleep efficiency (%)	88.09 ± 6.28	84.83 ± 6.68	88.33 ± 1.77	92.56 ± 1.36	90.21 ± 3.14	<0.001
WASO (min)	45.49 ± 7.98	52.01 ± 9.64	48.03 ± 5.24	41.47 ± 3.48	39.44 ± 4.09	<0.001
GAD score	5.63 ± 0.32	5.90 ± 0.30	5.76 ± 0.18	5.53 ± 0.19	5.26 ± 0.10	<0.001
PHQ score	5.83 ± 1.71	7.20 ± 1.80	6.65 ± 0.86	5.35 ± 0.57	3.81 ± 0.79	<0.001

Values are expressed as mean ± SD. Group comparisons were assessed using one-way ANOVA. PSQI, Pittsburgh Sleep Quality Index; WASO, wake after sleep onset.

### Associations between DPI and sleep, inflammatory, and hormonal biomarkers

Linear regression analyses examined the associations of DPI with sleep parameters, inflammatory markers, gut permeability, and hormonal biomarkers. In crude models, higher DPI was positively associated with sleep duration, sleep efficiency, and melatonin levels, and inversely associated with PSQI scores, WASO, CRP, IL-6, cortisol, and zonulin, reflecting favorable profiles across multiple outcomes.

After adjustment for age, sex, BMI, and physical activity (Model 1), associations with sleep efficiency, PSQI, WASO, and CRP remained significant, while the strength of associations for sleep duration, cortisol, and melatonin was reduced. In Model 2, the direction of association for PSQI reversed from negative (crude β = −0.079) to positive (β = 0.022), and associations with sleep duration also reversed direction, indicating that dietary covariates substantially modify these relationships. This suggests that the beneficial crude associations may be attributable to overall dietary patterns rather than phytochemical content specifically. Associations with cortisol, melatonin, and zonulin were attenuated and no longer statistically significant after full adjustment. These findings indicate that the relationship between DPI and sleep parameters is robust, whereas associations with hormonal and gut permeability markers may be influenced by additional dietary and lifestyle factors (Table 4). After applying Bonferroni correction for 10 primary outcomes (*α* = 0.005), the following associations remained statistically significant in fully adjusted models: sleep efficiency, PSQI, and WASO. Associations with sleep duration, CRP, IL-6, LPS, zonulin, cortisol, and melatonin should be considered exploratory and hypothesis-generating, as they did not meet the corrected significance threshold.

**Table 4:** Linear regression analysis of dietary phytochemical index (DPI) with sleep parameters, sleep quality, inflammatory, gut permeability, and hormonal biomarkers.

Dependent variable	Crude β (95% CI)	Model 1 β (95% CI)	Model 2 β (95% CI)	*P*-value after Bonferroni*	Bonferroni significant	Interpretation
Sleep duration (min)	2.13 (1.86, 2.40)	−0.04 (−0.27, 0.20)	−0.14 (−0.27, −0.01)	0.039	No	Exploratory
Sleep efficiency (%)	0.46 (0.42, 0.50)	0.07 (0.04, 0.10)	0.038 (0.022, 0.054)	<0.001	Yes	Primary finding
PSQI (global score)	−0.079 (−0.089, −0.069)	0.016 (0.010, 0.022)	0.022 (0.017, 0.027)	<0.001	Yes	Primary finding
WASO (min)	−0.46 (−0.52, −0.40)	0.059 (0.022, 0.096)	0.083 (0.065, 0.102)	<0.001	Yes	Primary finding
CRP (mg/L)	−0.059 (−0.065, −0.054)	−0.003 (−0.006, 0.000)	0.002 (0.00, 0.004)	0.027	No	Exploratory
IL-6 (pg./mL)	−0.063 (−0.070, −0.057)	−0.004 (−0.008, 0.000)	0.002 (0.00, 0.004)	0.014	No	Exploratory
LPS (EU/mL)	−0.0084 (−0.092, −0.076)	−0.055 (−0.010, −0.001)	0.0018 (−0.001, 0.004)	0.141 (ns)	No	Not significant
Zonulin (ng/mL)	−1.03 (−1.13, −0.93)	−0.091 (−0.144, −0.038)	−0.032 (−0.078, 0.015)	0.185 (ns in Model 2)	No	Not significant
Cortisol (nmol/L)	−2.06 (−2.25, −1.87)	−0.26 (−0.36, −0.15)	−0.071 (−0.156, 0.013)	0.099 (ns in Model 2)	No	Not significant
Melatonin (pg./mL)	0.59 (0.53, 0.65)	0.050 (0.002, 0.098)	−0.011 (−0.038, 0.016)	0.434 (ns)	No	Not significant

Values are unstandardized beta coefficients (β) with 95% confidence intervals. Crude model: Unadjusted (DPI only). Model 1: Adjusted for sex, age, BMI, and physical activity. Model 2: Model 1 + further adjusted for protein intake, total energy intake, dietary fiber, and milk intake.

Statistical significance after multiple comparison correction: *Bonferroni correction for 10 primary outcomes: α = 0.05/10 = 0.005.

Associations with *p* < 0.005 remain significant after correction.

## Discussion

In this cross-sectional study of 675 adults with T2DM, higher DPI scores were associated with longer and more efficient sleep, lower sleep fragmentation, and reduced subjective sleep disturbances. Additionally, higher DPI scores corresponded to lower levels of inflammatory markers (CRP, IL-6, TNF-α), favorable oxidative stress profiles, and circulating hormone levels (melatonin, cortisol). In fully adjusted models meeting correction for multiple comparisons (Bonferroni α = 0.005), higher DPI showed robust associations with improved objective sleep continuity (sleep efficiency, WASO) and lower subjective sleep disturbances (PSQI). Associations with other outcomes including sleep duration, inflammatory markers, and hormonal measures did not survive correction for multiple testing and should be interpreted as exploratory.

These findings are consistent with prior studies indicating that plant-based dietary patterns can influence sleep quality and metabolic health. Observational research in general populations has linked higher intake of fruits, vegetables, and polyphenol-rich foods to longer sleep duration and improved sleep efficiency (3, 12, 15, 21). However, like our study, prior research cannot distinguish whether benefits arise from phytochemical compounds specifically or from the broader nutritional profile of plant-based diets, including fiber, micronutrients, lower energy density, and displacement of less healthful foods. Previous studies in T2DM populations have primarily focused on macronutrients or overall dietary patterns, with limited evaluation of plant food intake using metrics like DPI. Our results extend existing evidence by demonstrating that higher DPI scores are associated not only with subjective sleep measures but also with objectively measured parameters, such as sleep latency, wake-after-sleep-onset, and sleep efficiency, measured via multi-night wearable devices in individuals with T2DM. However, after full adjustment for dietary covariates, the positive association between DPI and sleep duration observed in crude analyses was attenuated and reversed (Model 2: *β* = −0.14), clearly indicating that the crude associations reflect overall dietary quality and energy intake patterns rather than phytochemical-specific effects. This pattern of reversal suggests that the beneficial crude associations are driven by the overall healthfulness of plant-based dietary patterns rather than phytochemical content per se. Animal studies have suggested that microbial metabolites including SCFAs may influence circadian clock gene expression through epigenetic modifications, though our single-timepoint hormone measurements cannot assess circadian alignment, phase, or rhythmicity in humans (5, 22). After full adjustment for dietary covariates, the positive association between DPI and sleep duration observed in crude analyses was attenuated and reversed (Model 2: β = −0.14), suggesting that the crude association may be confounded by overall dietary quality and energy intake patterns.

The inverse association between DPI and inflammatory markers aligns with the anti-inflammatory properties of plant-based dietary patterns. While polyphenols and carotenoids have been shown to modulate nuclear factor-kappa B (NF-κB) signaling and reduce cytokine production in experimental studies (23), our observational data cannot attribute these effects specifically to phytochemicals versus other components of plant foods (fiber, magnesium, omega-3 fatty acids from nuts/seeds) or overall dietary quality.

Our study also observed favorable associations between DPI and glycemic control, lipid profiles, and hormonal markers, supporting the broader metabolic benefits of phytochemical-rich diets in diabetes management. These results are in line with studies reporting improved insulin sensitivity, reduced oxidative stress, and lower cardiometabolic risk among individuals adhering to polyphenol- or phytochemical-rich dietary pattern (10, 11). A critical limitation is that the DPI reflects overall dietary quality, not specific phytochemical effects. This is evident in our data: higher DPI quartiles were strongly associated with greater intake of fiber, fruits/vegetables, whole grains, and legumes. Consequently, crude models showing DPI’s benefits on sleep—such as increased duration—completely reversed direction after adjusting for these dietary components. This reversal indicates the observed benefits were attributable to the general advantages of a high-quality, plant-rich diet (e.g., higher fiber, better macronutrients), not to phytochemicals per se. Therefore, while supporting dietary guidance for whole plant foods in T2DM, our study cannot isolate the role of specific bioactive phytochemicals. References in our manuscript to “phytochemical-driven” mechanisms should be interpreted as relating to phytochemical-rich foods overall, not to isolated compounds.

In our study, higher DPI was also associated with elevated SCFA concentrations (acetate, propionate, butyrate). Animal and *in vitro* studies have suggested that SCFAs produced through microbial fermentation of dietary fibers and polyphenols may influence sleep through several pathways, including modulation of inflammatory signaling, influence on circadian clock gene expression, and effects on neurotransmitter systems (24–26). However, this study cannot validate the proposed mechanisms because: (1) single hormone time points cannot assess circadian or HPA axis dynamics; (2) cross-sectional analyses cannot establish causality or directionality—observed indirect effects may reflect statistical artifacts rather than true mediation. Our findings indicate that a higher plant-based diet, greater microbial diversity, and better sleep co-occur in T2DM, but whether this is causal, reverse causal, or driven by shared metabolic factors remains unknown. Longitudinal studies with functional multi-omics are needed to determine mechanisms.

This study has several important limitations. The cross-sectional design precludes causal inference and cannot establish temporal precedence between diet and sleep; therefore, causal pathways cannot be demonstrated and reverse causation remains possible. DPI cannot isolate phytochemical-specific effects from overall dietary quality, as evidenced by association reversals for sleep duration and PSQI after full dietary adjustment, indicating that crude associations likely reflect broader dietary patterns rather than phytochemical content per se. Melatonin and cortisol measurements at a single fasting timepoint cannot characterize circadian phase, rhythmicity, or HPA axis dynamics, limiting conclusions about circadian regulation. FFQ-based dietary assessment may be subject to recall and social desirability bias despite validated instruments and trained interviewers. After Bonferroni correction for multiple testing, only sleep efficiency, PSQI, and WASO remained statistically significant; associations with inflammatory and hormonal markers should be considered exploratory. Generalizability is limited to adults with T2DM from a single geographic region in China. Despite adjustment for multiple covariates, unmeasured confounding by medication effects, psychosocial stress, sleep environment, genetic factors, and other dietary components cannot be excluded. These limitations constrain interpretation to cross-sectional associations between plant-based dietary intake and favorable sleep and metabolic profiles, with causal pathways and mechanisms requiring investigation through longitudinal studies and randomized controlled trials.

In this cross-sectional study of 675 adults with T2DM, higher DPI scores were associated with higher sleep efficiency, and lower inflammation. However, the cross-sectional design precludes causal inference, DPI cannot distinguish phytochemical-specific from overall dietary quality effects, and statistical decomposition analyses cannot establish biological mediation. These findings demonstrate co-occurrence of plant-based dietary patterns and favorable sleep-metabolic profiles in T2DM but cannot determine causality or mechanisms. Future longitudinal studies, randomized trials, and functional microbiome characterization are needed to establish temporal relationships and biological pathways linking diet, microbiota, and sleep in diabetes management.

## Data Availability

The raw data supporting the conclusions of this article will be made available by the authors, without undue reservation.
